# Dr. Anis Rassi (★September 15, 1929; †June 6, 2021)

**DOI:** 10.1590/0037-8682-0517-2021

**Published:** 2021-11-12

**Authors:** Anis Rassi, Alejandro O. Luquetti

**Affiliations:** 1 Hospital do Coração Anis Rassi, Cardiologia, Goiânia, GO, Brasil.; 2 Universidade Federal de Goiás, Hospital das Clinicas, Núcleo de Estudos de Doença de Chagas, Goiânia, GO, Brasil.

Anis Rassi was born in the city of Vianópolis (State of Goiás) on September 15, 1929. He was the son of Abrão Rassi and Mariana Rassi and was the 9^th^ out of 10 children. Anis is predeceased by his parents and all siblings. Four of his brothers (Alberto, Luiz, Raul, and Afrânio) were also physicians, all having graduated from the Faculty of Medicine of the then-named University of Brazil, also known as the “Faculty of Medicine of Praia Vermelha” (beginning with Alberto Rassi, in 1940). Anis was married in 1956 to Evelyn Gabriel Rassi (who died in 2019). They had five children: four sons, Sérgio, Anis Jr, Alexandre, and Gustavo, who are all physicians (the first three are cardiologists and Gustavo, is a pathologist) and a daughter, Maria Cristina, who is a psychologist. All of their children have married and given them 11 grandchildren (six of them are physicians) and three great-grandchildren. Anis attended primary school in his hometown, secondary school in Silvânia (State of Goiás), Ginásio Anchieta, and Goiânia (Colégio Estadual de Goyaz), and high school in Rio de Janeiro (Colégio Andrews and Colégio Juruena). He entered the Faculty of Medicine of the University of Brazil on his first entrance exam in 1948, graduating in 1953. In 1954, he specialized in cardiology, initially at the General Polyclinic of Rio de Janeiro (Service of Dr. A. A. Vilela), and later as a visiting fellow for more than 10 years at the 2^nd^ Medical Clinic of the Hospital das Clínicas of the Faculty of Medicine of the University of São Paulo (Professor Luiz V. Décourt Service), at the Sabbado D'Ángelo Cardiology Institute (Service of Drs. Hugo Felipozzi and Adauto Barbosa Lima), and at the Institute of Cardiology of the State of São Paulo (today the Instituto Dante Pazzanese de Cardiologia). 

**Figure:**
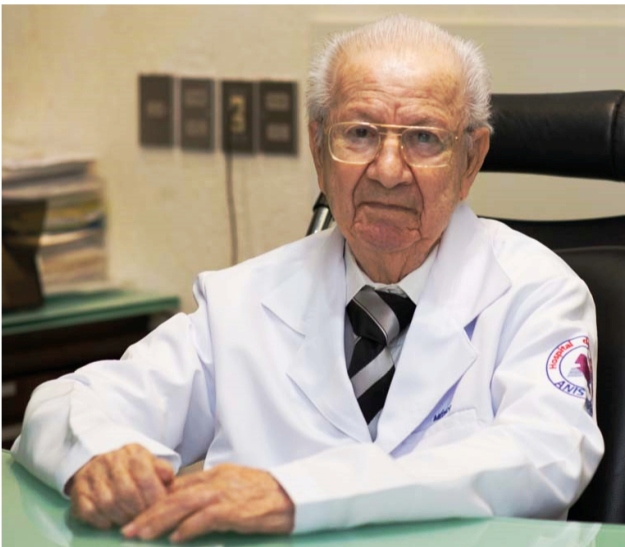

He began his professional practice in 1955, initially at Casa de Saúde Dr. Rassi (Goiânia), then at Hospital Rassi (currently State General Hospital of Goiânia Dr. Alberto Rassi), followed by Hospital São Salvador (Goiânia), and most recently at Hospital do Coração Anis Rassi, which he founded (April 27, 2003) and held the position of Director-President.

Since 1955, he had dedicated himself to the study of Chagas' disease, in its acute and chronic phases, with numerous original contributions. He was the author or co-author of 136 scientific articles on this disease. He had been published in national and international peer-reviewed journals and books, such as the New England Journal of Medicine^1^, Lancet^2^, Circulation^3^. He presented about 500 papers as an author or co-author, and participated in meetings in Brazil and abroad (USA, Mexico, Costa Rica, Venezuela, Colombia, Ecuador, Bolivia, Paraguay, Chile, Argentina, and Uruguay), with practically all of them on Chagas disease (general aspects, diagnosis, acute phase, arrhythmias of chronic heart disease, natural history, prognosis, symptomatic treatment, specific treatment, differential diagnosis, alternative mechanisms of transmission, and prevention of its transmission in blood banks).

 He was a consultant for the World Health Organization on specific treatment for Chagas disease. Anis was part of the Group of Founders of the Faculty of Medicine of the Federal University of Goiás (FUG) in the early 1960s. He was the head of the Department of Clinical Medicine of the Faculty of Medicine between 1962 and 1968. He participated in 22 examining committees or boards in Goiás and other states in Brazil. He received 44 laurels or awards, among which the following stand out: the Order of Merit Anhanguera in the degree of Grão Cavaleiro (Government of Goiás), honored guest of Sucre (Bolivia), the “Orden de Andrés Bello,” honor band class (Government of Venezuela), distinguished guest (Government of Paraguay), Emeritus Professor of the Faculty of Medicine of the FUG, eponymous of the Chagas Disease Outpatient Clinic of FUG/National Health Foundation, Hospital das Clínicas, and Distinguished Teacher Award from the Brazilian Society of Cardiology.

Certain characteristics of his personality deserve to be highlighted by those who accompanied him for over 40 years (one of us, ALO). Anis was extremely methodical, with clarity of reasoning and thinking far ahead of his time, possessing the innate qualities of true scientists. Since the beginning, he had a special interest in research on Chagas disease, perhaps because the disease was quite prevalent in his region and affected the most unfortunate. Matching his expertise with other researchers at the frequent meetings about Chagas disease, he saw the importance of keeping the patients' serum frozen (after their consent), for possible future studies, which he had been doing personally since 1975; he personally separated and stored the sera, with great zeal. At the same time, he became interested in xenodiagnosis, obtaining triatomines from different sources, and checking each box applied. The results were transferred to a few thousand of his patients' files. The results of his research were redacted and corrected repeatedly, usually late into the night. Anis was a tireless worker and we followed his work always with admiration for his perfectionism. He checked each reference, with extreme care, to see if it really was the first and most appropriate for the subject at hand. Certainly one of the few pioneers in the world interested in the specific treatment of acute and chronic Chagas disease, he started his work long before the appearance of nifurtimox, using different drugs that, unfortunately, did not show efficacy. In his obsession for checking drug efficacy, he accompanied each patient with dozens of blood samples and parasitological tests. We tried to follow his example of conserving the sera, which has been very useful for monitoring and verifying the cure of countless patients who still attend the outpatient clinic that bears his name. He had the brilliant idea of submitting hundreds of Chagas heart disease patients to a series of non-invasive tests and followed-up with them for several years; later, together with his siblings, he created a risk score (now known worldwide as the Rassi score, published in the New England Journal of Medicine^1^), which was capable of predicting the risk of death in these patients.

These patients had a particular affection for the master, sometimes coming from remote regions of the State of Goiás, Minas Gerais, and Bahia, for his free-of-charge evaluation. He retired as a professor; however, he remained active in his professional and research activities until recently. He died on June 6, 2021, at 91 years of age.

His is an exemplary life story that leaves its legacy to its medical children, grandchildren, and their students, among whom we are proud to be a part.

## References

[1] Rassi A, Rassi A, Little WC, Xavier SS, Rassi SG, Rassi AG (2006). Development and validation of a risk score for predicting death in Chagas' heart disease. N Engl J Med.

[2] Rassi A, Rassi A, Marin-Neto JA (2010). Chagas disease. Lancet.

[3] Rassi A, Rassi A, Rassi SG (2007). Predictors of mortality in chronic Chagas disease: a systematic review of observational studies. Circulation.

